# Tackling microbial threats in agriculture with integrative imaging and computational approaches

**DOI:** 10.1016/j.csbj.2020.12.018

**Published:** 2020-12-29

**Authors:** Nikhil Kumar Singh, Anik Dutta, Guido Puccetti, Daniel Croll

**Affiliations:** a Laboratory of Evolutionary Genetics, Institute of Biology, University of Neuchâtel, CH-2000 Neuchâtel, Switzerland; b Plant Pathology, Institute of Integrative Biology, ETH Zurich, CH-8092 Zurich, Switzerland; c Syngenta Crop Protection AG, CH-4332 Stein, Switzerland

**Keywords:** Agriculture, Plant pathogens, High-throughput phenotyping, Image analysis, Genome-wide association mapping, Sustainability

## Abstract

Pathogens and pests are one of the major threats to agricultural productivity worldwide. For decades, targeted resistance breeding was used to create crop cultivars that resist pathogens and environmental stress while retaining yields. The often decade-long process of crossing, selection, and field trials to create a new cultivar is challenged by the rapid rise of pathogens overcoming resistance. Similarly, antimicrobial compounds can rapidly lose efficacy due to resistance evolution. Here, we review three major areas where computational, imaging and experimental approaches are revolutionizing the management of pathogen damage on crops. Recognizing and scoring plant diseases have dramatically improved through high-throughput imaging techniques applicable both under well-controlled greenhouse conditions and directly in the field. However, computer vision of complex disease phenotypes will require significant improvements. In parallel, experimental setups similar to high-throughput drug discovery screens make it possible to screen thousands of pathogen strains for variation in resistance and other relevant phenotypic traits. Confocal microscopy and fluorescence can capture rich phenotypic information across pathogen genotypes. Through genome-wide association mapping approaches, phenotypic data helps to unravel the genetic architecture of stress- and virulence-related traits accelerating resistance breeding. Finally, joint, large-scale screenings of trait variation in crops and pathogens can yield fundamental insights into how pathogens face trade-offs in the adaptation to resistant crop varieties. We discuss how future implementations of such innovative approaches in breeding and pathogen screening can lead to more durable disease control.

## 1 Introduction

Feeding the world population requires a stable production of safe food, which is threatened by factors such as climate change, land degradation and diseases. Pathogens and pests cause significant reductions in agricultural productivity worldwide, and control strategies remain ineffective for many pathogens [1], [2], [3]. Around 20–30% of the global harvest is lost to plant diseases with additional post-harvest losses of up to 12% [4]. The two main approaches to protect crops from diseases are the deployment of resistant cultivars often carrying specific loci conferring resistance (*i.e.* R genes) [5] and the application of chemical compounds (*e.g.* fungicides, insecticides) [6], [7], [8]. Targeted resistance breeding has been practiced for decades to create cultivars that resist pathogens but retain desirable agricultural traits. Yet even modern molecular breeding techniques often involve multiple years of crossing, selection, and testing to create a new cultivar [9]. At the same time, plant pathogens often rapidly overcome resistance gene mediated immunity defeating expensive breeding efforts [10], [11]. Similarly, the deployment of synthetic chemicals is often followed by the rise of resistant pathogen populations [12], [13]. Tackling food security risks posed by pathogens will require bringing together fundamental insights into the biology of diseases, advancements in high-throughput approaches and innovations in computational tools.

Sustainable agricultural management practices critically rely on an understanding of the biology and evolutionary potential of the major crop pathogens. Emerging or re-emerging pathogens can only be contained by investigating their origins and migration routes, as well as their potential to counter-adapt to deployed control measures. Advancements in genomics, transcriptomics and proteomics have been instrumental to elucidate these questions and unravelled a broad range of molecular mechanisms governing host-pathogen interactions leading to more effective control strategies [14], [15], [16], [17]. Gene editing and breeding focused on the exploitation of natural genetic variability provide critical resources for introducing novel alleles into crop improvement efforts [18], [19]. Genetic crop improvement requires though large-scale screening of thousands of lines grown under different environmental conditions [20], [21]. On the pathogen side, the rapid identification of emerging resistance against chemicals or virulence on previously resistant crops is of utmost importance [3], [22]. Genotyping of plants and pathogens has reached impressive throughput at low cost, yet equivalent improvements in high-throughput screening of phenotypic information are largely lagging behind [20], [23]. This in turn has created a bottleneck for speed breeding efforts and automated monitoring of the plant health status during agricultural production. To highlight recent developments that have the potential to remedy these shortcomings, we first discuss crucial components of host-pathogen interactions that can be targeted. We then review recent developments in disease-focused plant phenomics enabled by high-throughput phenotyping platforms. Following that, we highlight advances in the screening of pathogen populations to detect early signs of resistance evolution or increased virulence. We argue that combining the above-mentioned approaches has yielded impressive insights into the genetic architecture of crop diseases. Finally, we discuss how innovative approaches can lead to more durable disease control.

### 1.1 Knowing the enemy: how pathogens interact with plants to cause disease

**Fig. 1:**
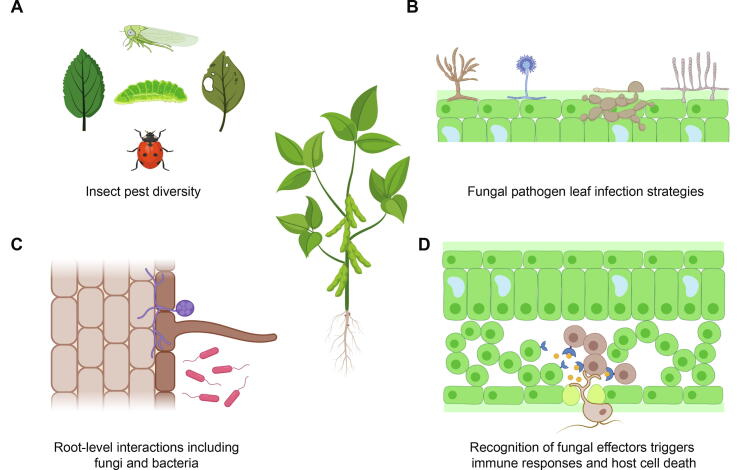
The biology of plant-pathogens interactions. A) A wide range of insects feed on leaves as herbivores. B) Longitudinal root section and rhizosphere. Underground interactions can have significant effects on the stem phenotype and on the entire plant. Filamentous fungi and bacteria can have beneficial effects on the plant by facilitating the acquisition of phosphorus and nitrogen or detrimental effects when confronted by root pathogens. C) Dorsiventral section of a leaf colonized by different pathogenic filamentous fungi. From the left: fungi of the *Fusarium*, *Aspergillus*, *Magnaporthe* and *Podosphaera* genus. D) Filamentous fungi and oomycetes causing leaf infections. Filamentous pathogens can penetrate into the mesophyll through stomata. Once inside the mesophyll, pathogens colonize the entire tissue within days or weeks. Depending on the lifestyle (biotrophic or necrotrophic), pathogens secrete small proteins (called effectors) to manipulate plant cells. Plants detecting effectors (blue receptors) can mount a hypersensitive response (HR) leading to an autophagy-like cell death prevents the spread of the pathogen. (For interpretation of the references to color in this figure legend, the reader is referred to the web version of this article.)

Major crop diseases are caused by pathogens including fungi, bacteria and viruses (Fig. 1). In addition, insect pests also cause substantial losses in agricultural production [24], [25]. Spores of fungi and bacteria can be transmitted by wind, rain splash and animals including humans and insects [26], [27]. Some pathogens penetrate plant tissues (Fig. 1B) with the help of vectors and then are surrounded by cytoplasm, cell membrane, or cell walls [28], [29]. In other cases, the pathogen makes contact with the external surface of the plant, and then deploys a penetration mechanism which can include highly specialized structures (*e.g.* appressoria) [30]. After invasion of the host tissue, the success of a pathogen on a specific host is largely explained by an interaction of gene products encoded in the host and pathogen genomes [31], [32]. Resistance (R) genes in plants encode proteins to directly or indirectly detect the presence of pathogen effectors (also known as avirulence factors or Avr) and trigger strong immune responses (Fig. 1B-D). Pathogens can escape recognition through sequence diversification or deletion of effector genes [33]. Despite the importance of single loci controlling the infection outcome, pathogenicity can have a complex and largely quantitative genetic basis [34], [35], [36], [37], [38], [39]. To successfully infect plants, pathogens also need to tolerate a series of abiotic stress factors (Fig. 1C). Since pathogen species have an optimal range of environmental conditions (*i.e.* temperature, pH, humidity) to thrive, changes in the environment can alter the pathogen's ability to cause damage [40]. Environmental factors such as annual temperature fluctuations, the quantity and pattern of precipitation, levels of CO_2_ and ozone can affect plant disease severity [2], [41]. Furthermore, the efficacy of pesticides can be significantly altered by weather conditions (*e.g.* wash-off of fungicides following strong rainfall). Hence, the challenge to contain pathogen damage in agriculture is to predict the emergence of virulent strains and the rise of fungicide resistance.

**Fig. 2:**
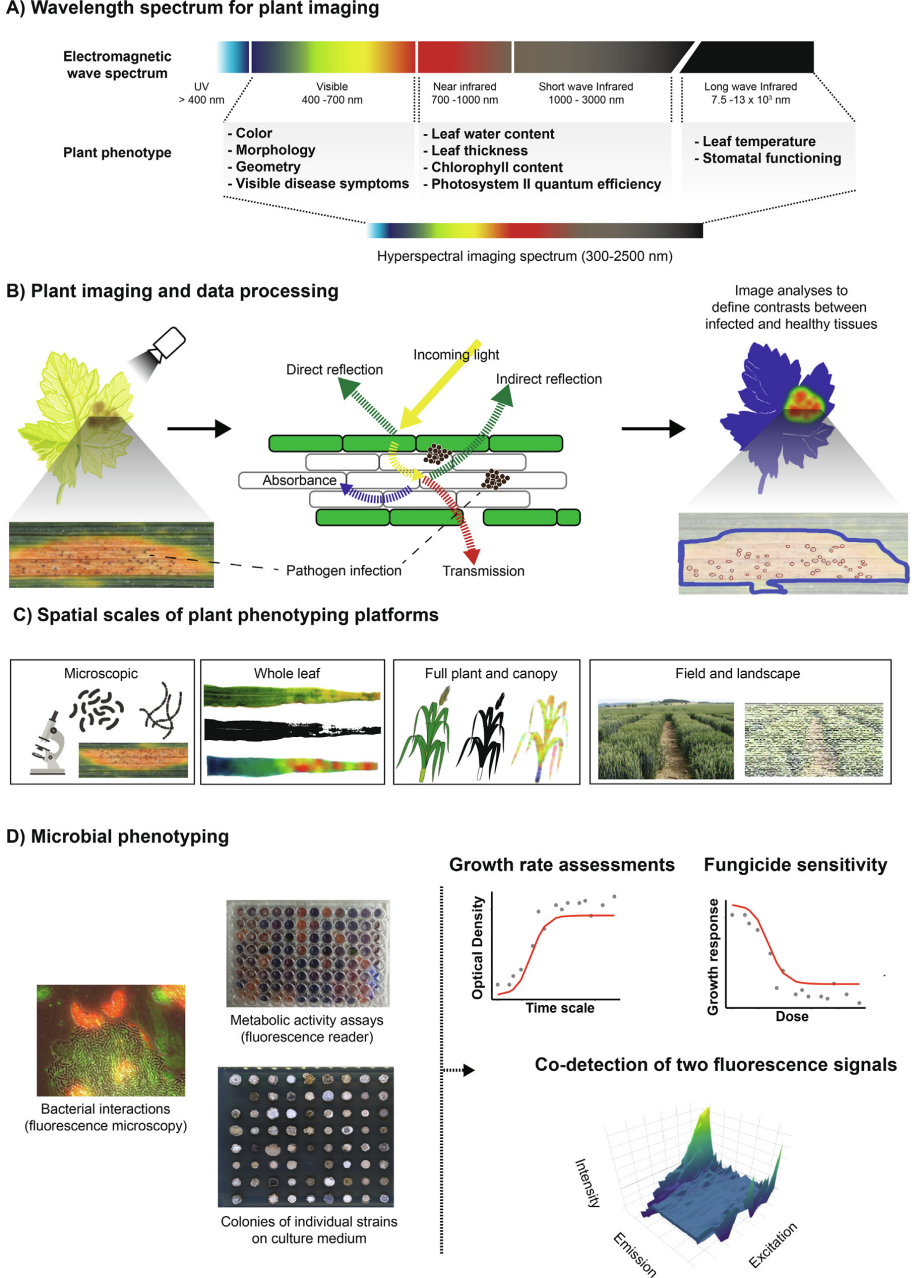
High-throughput phenotyping techniques for plants and pathogens. A) Light in the visible spectrum can be used to detect changes in color and morphology of infected plant tissue. Infrared and short-wave infrared enable to record changes in water content, leaf thickness and photosynthetic efficiency. Long-wave infrared allows assessments of plant surface temperatures. Hyperspectral sensors capture multiple images across the range of 300–2500 nm. B) Imaging systems assess absorption, transmission, or reflectance characteristics of the incident electromagnetic radiation interacting with the plant surface. Diseased plant tissue often differs in reflectance compared to healthy tissue. Image analysis algorithms define contrasts between diseased and non-diseased leaf areas. C) Spatial scales of plant phenotyping approaches. D) Screening of pathogen populations can be performed in liquid cultures or on solid media. The most common experiments monitor growth rates by assessing culture densities over time or estimate the dose–response curves when exposed to antimicrobial compounds. Co-cultures of multiple microbes may be analyzed using two distinct emission/excitation pairs specific for each the co-cultured species.

Given the possibly severe consequences, the early detection of resistance breakdowns in crops or loss of sensitivity to pesticides is critical. Detection early in the growing season and identifying previously uncharacterized pathogens remain major challenges. Classic plant disease diagnostics usually relies on visual symptom scoring by trained individuals categorizing disease severity on linear scales [42]. However, classic diagnostics is obviously only possible once symptoms have appeared [43]. Some pathogens (*i.e.* biotrophs) can feed on plant nutrients for a long period without causing apparent infection symptoms [44]. Pathogens killing plant cells to obtain nutrients are more obvious to detect [45]. Adding to the complexity of pathogen detection, many pathogens can undergo temporal shifts in infection lifestyles [46]. Plant infections often occur in patches with large areas of the field free of disease at an early stage of infestation. This is due to the short-distance dispersal of pathogens around the original disease foci [47], [48]. Hence, efficient, large-scale disease scoring is necessary to detect infections at early stages before the symptoms even become visible. Remote imaging and advanced data analysis can be used to identify disease foci and inform smart applications of chemical control agents (Fig. 2). The agricultural environment presents many complexities that are often poorly captured in greenhouse experiments. In fields, plants face fluctuating environmental conditions and are competing for light, water and nutrients. Hence, high-throughput phenotyping and analytical tools are largely subdivided into field-applicable approaches and laboratory/greenhouse setups.

Disease symptoms may include any range of changes in the color, shape or functioning of the plant as it responds to a pathogen and can be visualized at specific wavelengths (Fig. 2A). Depending on the pathogen, the disease symptoms can range from leaf spots, chlorosis, necrosis, wilting or overgrowth [49], [50]. However, plant stress beyond infections can activate protective mechanisms leading to suboptimal growth, chlorophyll loss or changes in surface temperatures [51], [52]. Such changes produce detectable shifts in the spectral signature compared to a healthy plant and can be measured by different methods (Fig. 2A-B, Table 1) [53]. Imaging systems rely on the quantification of absorption, transmission, or reflectance characteristics of the electromagnetic radiation interacting with the plant surface (Fig. 2A-B) [54]. We provide a set of specific disease-related phenotypes, which can be captured by different camera sensors across the wavelength spectrum in Table 1. For example, in the shortwave infrared spectrum (1000–3000 nm) water and biomolecules show characteristic reflectance patterns (Fig. 2A). A shortwave infrared sensor can detect an infected region based on differences in water content due to the disease (Fig. 2B). Computational algorithms can then highlight contrasts between infected and healthy areas to estimate disease severity at the leaf level (Fig. 2B). The various algorithms available for plant phenotype assessments have recently been reviewed in more detail [55]. Disease phenotype scoring is also largely dependent on the experimental setup. Plant disease phenotyping under greenhouse or growth chamber conditions largely eliminates fluctuations due to the environment and experiments can easily be replicated for the same genotypes. Such end-point experiments also allow invasive assessments of pathogen colonization on the surface and inside plant tissues using cross-section imaging of entire organs (*e.g.* leaves, Fig. 2C), wide-field and confocal microscopy (Fig. 2C), as well as various polymerase chain reaction (PCR) and sequencing-based methods to estimate abundance. Such well-controlled measurements from mostly greenhouse experiments have been used to perform QTL mapping and genome-wide association studies (GWAS) to detect markers associated with resistance and inform breeding programs.

**Table 1:** Disease assessments enabled by high throughput sensors.

Image sensor type	Examples of measurable phenotypes	Disease/pathogens	Reference
RGB	• Color • Morphology • Biomass • Physiology • Disease symptoms • Germination rate	• Potato late blight • Citrus canker • Cercospora leaf spot • Sugarbeet rust • Anthracnose • Septoria tritici blotch	[60], [89], [95], [180], [181], [182], [183], [184], [185], [186], [187]
Multispectral and hyperspectral sensors	• Nutrient status, • Water content • Senescence • Photosynthetic efficiency	• Laurel wilt • Powdery mildew • Cercospora leaf spot • Rusts • Fusarium Head blight	[67], [93], [94], [188], [189], [190], [191], [192]
Thermal sensors	• Transpiration • Heat stress • Senescence • Leaf/canopy temperature	• Downy mildew • Wilt • Scab disease	[193], [194], [195]
Infrared sensors	• Phenotypes measured with thermal and hyperspectral sensors • Leaf water content • Photosynthetic efficiency	• Powdery mildew • Net blotch • Brown rust • Fusarium Head blight	[51], [190], [196], [197], [198], [199]

### 1.2 High-throughput plant health assessment under field conditions

Technologies suitable for screening plant diseases even in early infection stages have become widely deployed [20]. Phenotyping platforms integrating such technology allow both proximal and remote monitoring of single plant leaves (or other organs), individual plants as well as entire fields (Fig. 2C). Capturing plant phenotypes under field conditions consists either of measurements from space or the air with cameras mounted on satellites (WorldView-3, www.digitalglobe.com), unmanned aerial vehicles (UAVs), parachutes, blimps, manned rotocopters or fixed-wing systems [20], [56], [57]. Satellite imaging can provide multi-spectral images with a resolution ranging from meters to hundreds of meters [57]. However, there are major limitations due to weather conditions, the frequency of image capture, and overall costs. Satellite imaging is also most useful for regional or continental scale assessments of vegetation cover rather than crop breeding trials in individual fields. Recent improvements in high-resolution satellite imagery, *e.g.* GF-1 from China or SPOT from Europe, can provide time-resolved phenotyping of individual fields at the meter scale [58], [59]. Such resolution is able to capture transitions in reflectance due to senescence or pathogen outbreaks. Unmanned aerial vehicles (UAV) have become popular in recent years because of the ease of deployment and possibilities to mount high-resolution image capture systems [56], [60]. A range of sensors can be installed on UAVs depending on the payload capacity and type of data required. Based on the spectrum and number of bands, the sensors can be classified into several types including visible light (RGB) [61], [62], [63], multispectral, hyperspectral [64], [65], [66], [67], thermal infrared [68], [69] and 3D laser imaging [70], [71]. As an application example, a commercialized quadrotor UAV mounted with digital and multispectral cameras enabled the detection and monitoring of rice sheath blight [66]. A hexacopter mounted with RGB cameras enabled wheat plant height measurements matching well manual and ground-based LiDAR sensor measurements [72]. Monitoring the progression of late blight disease in a potato field was possible using an aerial RGB camera [60]. Thermal infrared sensors have also been successfully used to quantify plant responses towards drought stress in Black Poplar [73], ground cover in sorghum, canopy temperature in sugarcane and crop lodging in wheat [74]. A major limitation of aerial camera based phenotyping is errors introduced while generating orthomosaics, which are composite images of stitched and geometrically corrected photographs [57], [75]. Inaccuracies in orthomosaics can be introduced by low camera sensor resolution, altitude and how the camera positioning was determined during capture [76]. However, improvements in rectification algorithms and drone technologies have reduced orthomosaic errors significantly [77]. Orthomosaic inaccuracies can also be remedied by supplementing imagery captured by proximal phenotyping platforms [78], [79].

### 1.3 Proximal plant phenotyping

Proximal phenotyping is mostly deployed in greenhouse experiments or in well-controlled field experiments. Sensors covering a broad range of spectra can be mounted on stationary platforms or, for outdoor use, on suspension cables, robots and tractors [20], [23], [80], [81], [82], [83], [84]. A fixed framework over a large field has advantages over both vehicle- and UAV-based phenotyping and is not limited by sensor payloads and battery capacity [85]. For example, the Field Phenotyping Platform (FIP) established at the ETH Zurich uses a suspended cable setup to move sensors over a large experimental field site [84]. Similar setups exist at the University of Nebraska to phenotype maize and soybean cultures [86]. These proximal phenotyping platforms benefit from having multiple mounted sensors capturing phenotypic trait information over a wide range of wavelengths. For example, a proximal hyperspectral sensor platform was capable to detect powdery mildew infections based on barley canopy surveys only [67]. Imaging based on fluorescence is often used for proximal phenotyping to quantify phenotypic changes related to photosynthesis, nitrogen content and diseases like Septoria tritici blotch (Fig. 2A-B) [42], [87], [88], [89]. Proximal phenotyping platforms can measure ﻿canopy temperature, nitrogen content, as well as phenotypic traits including leaf area and plant height [90]. Such efficient, whole-plant phenotyping helped accelerate the selection of drought-resistant rice plants [89]. The platform was designed for imaging in the visible spectrum to record morphological features. The system also included infrared and near-infrared imaging to quantify water content, as well as temperature and fluorescence measures to quantify photosynthesis efficiency [89]. ﻿The platform could accurately distinguish tolerant and susceptible plants and thus enabling rapid selection for speed breeding. Downstream applications included functional genetic studies for drought tolerance and GWAS applications, which in turn can feed into marker-assisted breeding efforts. In a different application, chlorophyll fluorescence and thermal (infrared) phenotyping of ~300 Iranian wheat cultivars combined with GWAS revealed adaptive alleles for drought stress useful for marker-assisted selection [91].

Current plant phenotyping technologies and image processing algorithms struggle to reliably differentiate disease symptoms originating from multiple or unknown pathogens [92], [93]. This is partially due to the complexity of fungal and bacterial disease symptoms on crops, but is also due to the fact that disease symptoms during early infection stages are hardly diagnostic for specific pathogens. Nutrient or water deficiencies (in the absence of pathogens) can produce symptoms that are difficult to differentiate from disease phenotypes. Molecular detection methods (*i.e.* qPCR) are often required to clearly establish what is likely to cause a disease. Interesting developments to overcome these limitations are *e.g.* the differential reflectance spectra (400–1050 nm) of sugar beet leaves, which helps distinguish three different fungal pathogens including *Cercospora beticola*, *Erysiphe betae*, and *Uromyces betae* [94]. Combined thermal and visible light image data was fed to a machine-learning algorithm to differentiate infections by the tomato powdery mildew fungus *Oidium neolycopersici* [95]. One major limitation of plant disease high-throughput phenotyping is the timeframe of disease detection. Imaging which relies on reduced photosynthesis as a proxy for disease progression can only detect symptoms once the pathogen invades the plant tissue and reduces chlorophyll activity (Fig. 1, Fig. 2). Hyperspectral or multispectral sensors can distinguish diseased leaves from healthy ones much earlier than imaging in the visible spectrum only. Abdulridha et al. [96] were able to detect and distinguish the onset of target spot caused by the fungus *Corynespora cassicola* and bacterial spot caused by *Xanthomonas perforans* on tomato in the asymptomatic phase. The multi-spectral imaging approach was successful both under laboratory and field conditions. Such robust phenotyping methods could make it possible to detect infection foci in the field. Emerging infections could then be isolated and treated individually by the deployment of specific fungicides.

Plant phenotyping technologies have generated data to populate public image databases (summarized in [97]). Such data can be used for machine learning and shows promising results in predicting disease onsets and identifying the pathogen species [95], [98], [99]. Using a public dataset of 54,306 images of healthy and diseased plant leaves, a deep learning network could identify 26 different diseases with 99.35% accuracy [99]. The advancement of machine learning applied to the problem of plant phenotyping has been limited mostly by two factors: i) the limitation of publicly available image databases of crop disease phenotypes under a variety of environmental conditions and ii) poor annotation of image data and the captured symptoms [100], [101]. The lack of robust training datasets has forced researchers to use suboptimal databases leading to ambiguity in distinguishing disease phenotypes from senescence or other environmental factors [99], [100], [102]. Thus, despite the enormous potential of machine learning for plant disease scoring, the current implementations will need to be improved with large reference datasets.

### 1.4 Pathogen screenings to improve the durability of plant resistance

Plant-microbe interactions are often subject to rapid co-evolutionary dynamics altering the genetic make-up of both the host and the pathogen [103]. Evolving pathogens make breeding for resistance obviously challenging. In addition, outbreaks of new pathogens have significantly increased [104] including *e.g.* recent outbreaks of *Magnaporthe oryzae* and *Puccinia graminis* f. sp. *tritici* in Bangladesh and Italy, respectively, endanger wheat production [104], [105], [106], [107]. Rapid change in crop pathogen populations is most evident through the rise of fungicide resistance [108], [109] but many other phenotypes show high variability as well [110], [111]. Some pathogens gained virulence traits by acquiring genetic material through horizontal transfer. A striking example includes the gain of virulence on wheat by transferring a key toxin-encoding gene between fungal species [112]. Among the most diverse pathogens is the cosmopolitan fungus *Zymoseptoria tritici* attacking wheat. Cultivar resistance has been largely broken down and some new virulence arose within the span of a few years [35], [113]. *Z. tritici* populations are genetically and phenotypically highly diverse with intra-field diversity approaching levels of diversity at the continental or global scale [114], [115], [116]. To counter highly diverse and rapidly evolving pathogens, breeding programs often attempt to prevent resistance breakdowns by combining (‘pyramiding’) several resistance genes [35], [117].

Despite the challenges associated with rapidly evolving pathogen species, heritable trait variation can be exploited to identify mechanisms underlying virulence [38], [118]. A number of major crop pathogens are readily culturable under sterile laboratory conditions allowing reproducible assessments of trait expression. The clonal propagation in sterile medium allows the efficient replication of phenotypes expressed by different genotypes. A major application of high-throughput pathogen screenings are measurements of fungicide sensitivity, which is relevant for the early detection of resistance mutations at the regional or continental scale [119]. More broadly, stress response assessments of fungal and bacterial pathogens can provide important clues about the expression of virulence factors, multi-drug resistance, biofilm formation, and antimicrobial resistance [117], [120]. This is because stress induced in *in vitro* setups can share similarities with infection stress conditions. During infection stages, pathogens have to cope with various host defense responses including nutrient deprivation, pH variation, *etc*. Hence, *in vitro* conditions can be useful triggers to express virulence-related proteins. Beyond measuring growth rates as a proxy for the physiological state, phenotypic screens can be extended to measurements of spore shape heterogeneity [121], cell viability, as the percentage live cells, or cell vitality, defined as the physiological capabilities of a cell [122], [123] and can be assessed with flow cytometry and fluorescence readouts. More detailed analyses focus on variation in the infection life cycle on the host [124]. Finally, assessing temperature adaptation can provide important clues about the adaptive potential and the ability of pathogens to cope with future climates [124], [125]. However, manual handling procedures will need to be replaced by robotization to improve throughput.

High-throughput phenotyping platforms for microbial organisms have dramatically advanced in automating cell culturing, liquid handling, spotting on culture medium as dense arrays and integration of fluorescence measurements and microscopy [126], [127], [128], [129]. Such technologies were often first applied for active compound screens in drug discovery programs of the pharmaceutical industry [130]. In the last years, several pipelines have been developed for high-throughput *in vitro* screening to investigate drug tolerance genes and to elucidate aspects of pathogenicity [128], [129]. High-throughput pipelines benefit from robotics, such as Rotor-HDA, which can duplicate and back up large strain libraries in solid or liquid media in the format of 96–6144 isolates per plate. Growth and fungicide susceptibility can be assessed using optical density (OD), colony size, or the fluorescence of liquid cultures (Fig. 1D) [131]. Moreover, culture colors are powerful proxies for the production of melanin or other secondary metabolites [132]. Other high-throughput platforms incorporate multiplexed microfluidic cell culture, automated programmable fluid handling for cell perturbation, quantitative time-lapse microscopy, and computational analysis of time-lapse movies [133]. Such platforms are most useful for perturbation experiments (*e.g.* osmotic shock or exposure to a drug) with a fluorescent readout sensitive to changes in gene expression or subcellular localization. The main goal of the technology is to accelerate drug discovery by screening large antimicrobial compound libraries at a rate of thousands of compounds per week. Efforts are as well made in the development of high-throughput approaches to generate mutated versions of drug targets [128]. High-throughput screening methods are further used to characterize metabolic, pharmacokinetic and toxicological data about new drugs and reduce the costs of antimicrobial compound development [129], [134]. During the development of a new pesticide, field trials can be extremely costly. Therefore, high-throughput *in vitro* screening of diverse pathogen population scans is very informative about possible standing resistance, which can prevent rapid efficacy failures under field conditions [135].

The wealth of information on phenotypic trait variation in pathogen species combined with low-cost sequencing can be exploited for GWAS [136]. Since the debut of the technique in 2005, GWAS revealed hundreds of thousands of single nucleotide polymorphisms (SNPs) and structural variants associated with thousands of different phenotypes mainly focused on humans [136], [137], [138]. Plant pathogens populations often harbor both virulent and avirulent strains. Hence, with reliable and robust phenotyping, GWAS analyses can identify genes underlying specific gains in virulence. Mapping populations of ~100 strains were sufficient to identify key virulence factors [139], [140], [141] and fungicide resistance loci in fungal pathogens [135], [139], [140], [142], [143]. Genetic mapping studies can also inform the development process of new fungicides by the early identification of resistance “hotspot” genes [135]. A critical factor for successful GWAS applications is the availability of high-quality genomic resources representing the genetic diversity of the pathogen species. Recent pangenome analyses of plant pathogens have laid the foundation for such work [144].

### 1.5 Deciphering complex host-pathogen interactions – a way forward

The application of imaging combined with computational techniques enabled enormous progress in breeding resistant crops and detecting the emergence of new pathogen threats. To reduce complexity, most studies until now focused either on variation on the plant or the pathogen side. However, the nature of host-pathogen interactions can vary across space and time [145], [146]. In addition to such genotype-by-genotype interactions, environmental conditions (humidity, temperature, competing microbes, *etc.*) influence the outcome of infections. The complexity arises in part from complex pathogen infection cycles starting from initial host contact to transmission to new hosts [147], [148]. Each infection stage is likely governed, to some extent, by distinct genes. Improving our abilities to predict infection outcomes across many different plant-by-pathogen interactions and environments is therefore a critical step in improving crop resistance and controlling disease outbreaks in the long term.

**Fig. 3:**
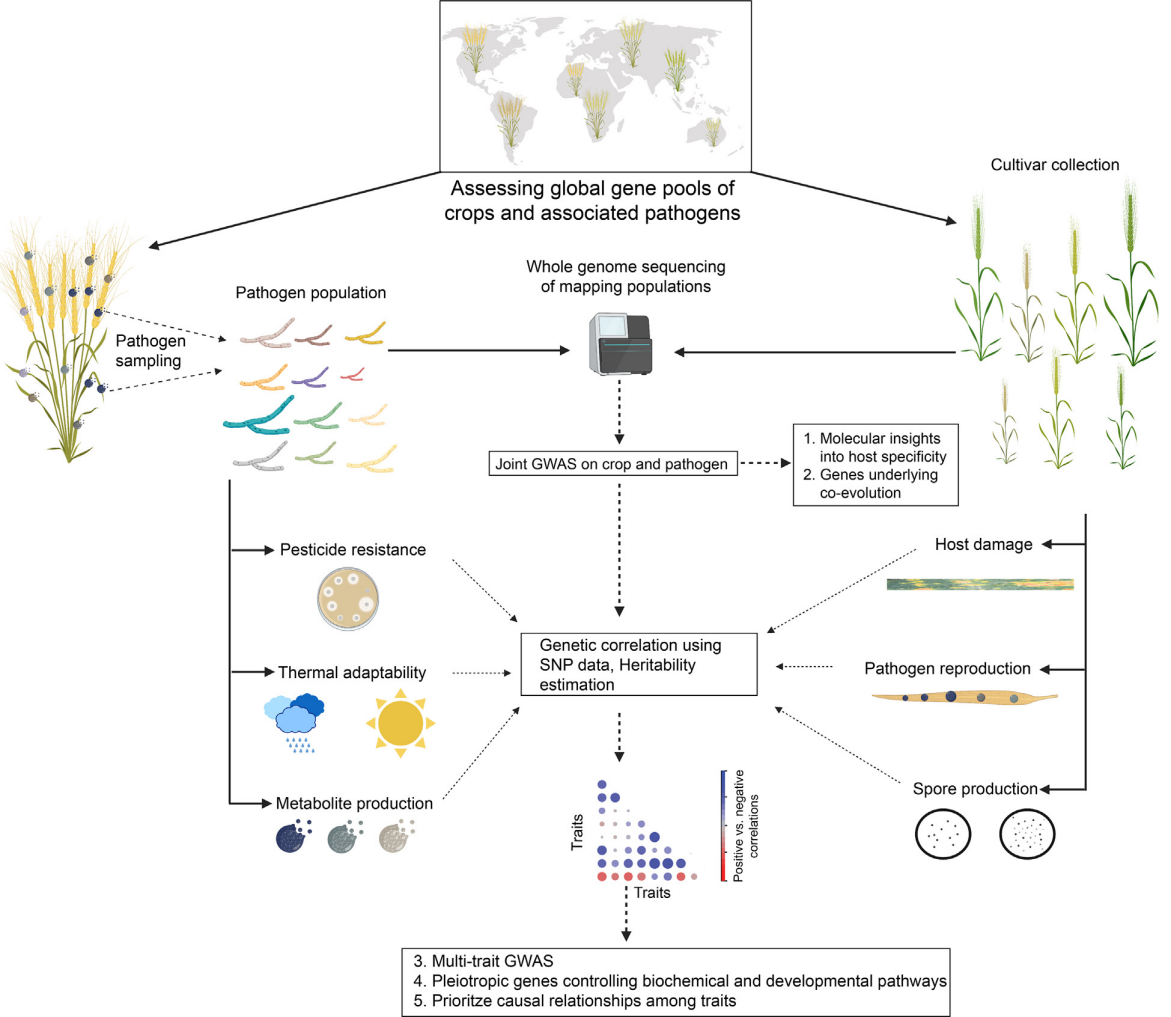
A comprehensive framework for determining the genetic basis of crop-pathogen interactions. Genetically diverse pathogen populations and crop cultivars from different geographies form the basis of the screening. Genome sequencing enables to conduct joint genome-wide association studies (GWAS) to determine the genetic architecture of virulence (in pathogens) and resistance (in crops). Global populations of both pathogen and crop will capture most relevant genetic variation. Beyond virulence, pathogen populations can be screened for loci underlying pesticide resistance, thermal adaptation and metabolite production (*e.g.* melanin). Identifying genetic correlations among pathogen traits facilitates the identification of pleiotropic genes governing trade-offs. Some illustrations were provided by biorender.com according to their usage conditions.

Integration of different *omics* datasets to build biological networks (*e.g.* gene co-expression or protein–protein interaction networks) has become a powerful approach to unravel genetic factors controlling biological interactions [149], [150], [151]. Beyond this, innovative applications of GWAS can help to identify causal genes by studying host and pathogen genotypes simultaneously in an infection matrix [152], [153] (Fig. 3). Applying such an approach to the *Arabidopsis thaliana* - *Xanthomonas arboricola* interaction, Wang et al. [152] identified specific genes for quantitative disease resistance in the host that are effective only against a specific set of pathogen strains. Studying crop diseases as an infection matrix can help to identify genomic regions underlying host-pathogen co-evolution and genes responsible for specific phenotypes [154], [155], [156]. However, applications in the agricultural context are largely missing for now as such approaches are experimentally demanding and costly. Improved pathogen inoculation techniques such as detached leaf assays [157], [158], [159] or head assays [160] combined with automated image analysis [161] will reduce the experimental burden. Consequently, collecting precise phenotypic data from a large infection matrix of hosts and pathogens has become increasingly accessible [157]. Beyond increased sample sizes of the analyzed host and pathogen genotypes, special care is required to adequately cover host and pathogen genetic diversity. Using a global set of populations of the major wheat pathogen *Z. tritici*, a large set of loci associated with pathogen virulence and reproduction on different hosts were identified [38], [162]. A large infection matrix of 98 strains of *Botrytis cinerea* and 90 plant genotypes of eight species revealed a highly polygenic architecture of pathogen virulence and host specialization [163]. Hence, simultaneously extending genetic diversity screens of the host and pathogen provides a powerful approach to comprehensively map the genetic architecture of virulence and resistance. Particularly relevant pathogens to investigate are the ones capable of infecting multiple plant organs (*e.g.* blackleg in canola) or at different growth stages. Bigger sample sizes in both host and pathogen will also improve heritability estimates (*e.g.* as shown in human studies [164]). Overall, expanding the breadth of host and pathogen genetic diversity in screenings will help to identify previously unknown resistances/susceptibilities in crop germplasm.

### 1.6 Exploiting pathogen weakness driven by trade-offs

How successful pathogens cope in diverse environments depends on life-history trade-offs, which arise from resource allocation dilemmas and antagonistic gene actions. A trade-off indicates that an increase in one trait is associated with a decrease in another trait. Such trade-offs are typically dependent on the host genetic background and abiotic conditions. Some pathogen strains have evolved specialization on certain hosts or climatic conditions to maximize their performance. Classic examples include the wild pathosystem of *Plantago lanceolata*-*Podosphaera plantaginis* interactions [165], [166]. Trade-offs are likely a key factor maintaining polymorphisms in pathogen populations [167], [168]. Studies have already identified trade-offs between foliar damage and asexual transmission [169], sporangia size and number [170], latency in spore production, size and sporulation rates [171]. Analyses of demethylation inhibitors (DMI) fungicide-sensitive and resistant isolates of the sugar beet pathogen *C. beticola* have shown that resistant isolates have significantly lower virulence and spore production than sensitive isolates [172]. No differences were found for incubation periods, mycelial growth, germination of conidia and germ tube length. Similarly, in the bacterial pathogen *Ralstonia solanacearum* mutants lacking the gene for synthesizing an exopolysaccharide virulence factor show increased growth rates compared to the wild-type strain [173], [174]. Besides, agricultural pathogens likely face many additional, yet unknown trade-offs because pathogens must also survive outside of annual crop hosts [175], [176]. The genetic basis of trade-offs remains largely unknown hindering the exploitation of inherent pathogen weaknesses. This is partly because trade-offs are generally expressed by phenotypic trait correlations, which are often confounded by environmental variation and genetic substructure. Identifying genetic correlations among traits can circumvent the above challenges as it reflects the direct effect of genetic factors controlling trade-offs and are robust to confounding factors [177]. A recent study investigated genetic trade-offs based on genetic correlations in the wheat fungal pathogen *Z. tritici.* Performance of a global strain collection on twelve wheat varieties and in various abiotic conditions revealed a broad pleiotropic control of pathogen performance on and off the host [162]. Weaknesses of pathogens in specific environments will inform more efficient designs of disease control and prevent future resistance breakdowns. Hence, comprehensive maps of genetic trade-offs will possibly enable innovative disease control strategies (Fig. 3). Beyond revealing trade-offs, correlated traits can be combined to perform multi-trait GWAS to pinpoint pleiotropic genes and determine causality among traits in specific environments [178], [179] (Fig. 3).

## 2 Summary and outlook

Technological progress in assessing susceptibility of large collections of crop plants to pathogen damage is crucial for modern resistance breeding efforts. A variety of image capture techniques allow to monitor plant damage at the cellular, leaf, whole plant or field level. Most applications focus on the visible spectrum but hyperspectral imaging platforms have recently gained the ability to detect pathogen infestation even before the appearance of symptoms. A major area of going research is to improve image analyses algorithms to detect and classify pathogen damage. Variation within individual pathogen species can be highly informative about the rise of new virulence or pesticide resistance. Robotics applied to automate the culturing of thousands of pathogen strains enables screening for metabolic variation, drug susceptibility and production of secondary metabolites improving our understanding how pathogens cope with the agricultural environment. Both high-throughput plant and pathogen phenotyping efforts can be combined with genome sequencing and GWAS applications. Unraveling the genetic basis of host resistance helps to speed up breeding efforts through marker-assisted selection. Analysis of pathogen populations can be informative about possible trade-offs in the emergence of virulence or pesticide resistance.

Future directions of research should focus on a set of complementary research areas.

- Create efficient pipelines merging imaging and molecular assays for pathogen detection. Such integrated systems could help farmers deploy appropriate counter-measures in the field prior to widespread damage and reduce overall pesticide application.

- The susceptibility of crop cultivars to major pathogens should be re-assessed continuously to detect changes in the virulence profile of the prevalent pathogens. Rapid evolution in pathogens can lead to catastrophic resistance breakdowns and must be detected early enough. High-throughput imaging systems capturing disease symptoms should be combined with machine learning to effectively recognize changes in virulence profiles. The lack of curated and open access disease image databases is currently slowing progress.

- Regional monitoring efforts of resistance breakdowns or the loss of pesticide efficacy can be achieved by high-throughput genomic screening of infected leaf material. Efficient genotyping assays focusing on major genes are likely to scale well to broad applications. Bioinformatic procedures for such genomic data analyses are largely in place. A successful implementation of such genomic monitoring will also help to detect the arrival of new pathogens early enough to deploy resistant cultivars or implement changes in pesticide application regimes.

- The systematic identification of trade-offs faced by pathogens adapting to pesticides and resistant crop cultivars could lead to more durable control measures.

## CRediT authorship contribution statement

**Nikhil Kumar Singh:** Conceptualization, Writing - original draft, Visualization. **Anik Dutta:** Writing - original draft, Visualization. **Guido Puccetti:** Writing - original draft, Visualization. **Daniel Croll:** Conceptualization, Writing - original draft, Supervision, Funding acquisition.

## Declaration of Competing Interest

The authors declare that they have no known competing financial interests or personal relationships that could have appeared to influence the work reported in this paper.
